# Sex as a Moderator in the Association Between Rest-Activity Rhythms and Cardiometabolic Profiles

**DOI:** 10.1093/geroni/igaf122.489

**Published:** 2025-12-31

**Authors:** Chooza Moon, Meina Zhang

**Affiliations:** University of Iowa, Iowa City, Iowa, United States; UT Health, United States

## Abstract

Cardiometabolic profiles are the significant indicators of the development of cardiovascular disease in midlife and older adults. Rest-activity rhythm (RAR) reflects the pattern of physical activity, rest, and sleep throughout 24 hours. Although sex may be linked to both RAR and cardiometabolic profiles, limited research has examined the role of sex in the relationship between RAR and cardiometabolic profiles. We analyzed data from the Midlife in the United States Survey (MIDUS 2) (N = 440, N (female)= 266(61%), mean age male (SD)= 55.4 ( 3.5), mean age female (SD) = 53.2 (11.5) ) to examine the moderating role of sex on the relationships between RAR parameters (i.e., acrophase and R-square) and cardiometabolic profiles (i.e., HbA1C, Fasting glucose, LDL, total cholesterol, IL-6, C-Reactive Protein). RAR was measured using 7-day actigraphy. After controlling for age, we found the interaction between sex and R-square were significantly associated with HbA1C (β = -3.97, p = 0.003), fasting glucose (β =-101.90, p = 0.0038), total cholesterol (β = 121.75, p = 0.007), LDL (β = 87.94, p = 0.023), but not with IL-6 and C-Reactive Protein. However, the moderating role of acrophase and sex were not significant in any of the outcomes. Our findings also demonstrated a significant interaction effect between activity consistency and sex associated with cardiometabolic profiles. These findings suggest a potential moderation effect of sex on rest-activity rhythmicity and cardiometabolic profiles rather than on peak timing. Future studies should utilize more accurate and direct measures of cardiovascular function, along with direct circadian rhythm data, in larger prospective cohorts.

